# Delayed treatment seeking and its associated factors among people with schizophrenia spectrum disorders who are on follow-up at Dilla University Referral Hospital in the southern region of Ethiopia, 2022: a cross-sectional study

**DOI:** 10.3389/fpsyt.2023.1230448

**Published:** 2023-09-19

**Authors:** Misrak Negash, Bethel Temesgen, Chalachew Kassaw, Lulu Abebe, Solomon Moges, Yohanes Sime, Solomon Yimer

**Affiliations:** ^1^Department of Psychiatry, College of Medicine and Health Science, Dilla University, Dilla, Ethiopia; ^2^Department of Psychiatry, College of Medicine and Health Science, Woldia University, Woldia, Ethiopia; ^3^Department of Psychiatry, College of Medicine and Health Science, Wollo University, Dessie, Ethiopia

**Keywords:** delayed treatment seeking, schizophrenia spectrum disorder, factors associated, Dilla University Referral Hospital, southern Ethiopia

## Abstract

**Background:**

Delay in psychiatric treatment leads to increased morbidity and mortality, as well as the emergence of several psychiatric and physical comorbidities and the use of life-threatening and life-altering self-treatments (such as licit and illicit substance misuse). Delaying detection and taking preventive measures against its modifiable factors are crucial for a better prognosis.

**Objective:**

To assess delayed treatment seeking and its associated factors among people with schizophrenia spectrum disorders who are on follow-up at Dilla University Referral Hospital in the southern region of Ethiopia.

**Method:**

An institution-based cross-sectional study was conducted between 8 June and 11 September at Dilla University Referral Hospital in the southern region of Ethiopia in 2022. Epicollect was used to collect data from 414 randomly selected participants using an interviewer-administered questionnaire. Delayed treatment seeking was determined using participants' medical records and a semi-structured questionnaire. The data were analyzed using Statistical Package for Social Sciences (SPSS) version 26. A logistic regression analysis was conducted to identify the explanatory variables for delayed treatment.

**Results:**

The magnitude of delayed treatment seeking was 49.8% (95%CI = 44.9, 54.3). Study participants with disengaged family cohesion [AOR = 3.97, 95%CI = (2.999, 7.193)], inflexible family adaptability [AOR = 2.00, 95%CI = (1.686, 4.044)], who lack awareness about the availability of psychiatric treatment [AOR = 1.63, 95%CI = (1.362, 2.626)], high internalized stigma [AOR = 3.24, 95%CI = (2.770, 5.514)], and those with a negative attitude toward psychiatric treatment [AOR = 2.88, 95%CI = (2.034, 4.469)] had delayed seeking treatment. However, the participants whose educational status was higher than diploma [AOR = 0.040, 95%CI = (0.026, 0.077)] and high school [AOR = 0.09, 95%CI = (0.071, 0.204)] were less likely to have delayed seeking treatment.

**Conclusions:**

There is a significant delay in seeking modern psychiatric treatment. Stigma, a lack of awareness of where treatment is available, disengaged family cohesion, inflexible family adaptability, distance to a health facility >5 km, and a negative attitude toward psychiatric treatment were barriers to seeking appropriate care.

## Background

Globally, mental health has become an increasingly significant public health issue. However, compared to the rates for physical health conditions, the rates of mental health service utilization are still low (1). Many psychiatric patients do not receive appropriate treatment services at an early stage, which significantly affects the progression of the disease (2). A study conducted in Ethiopia revealed that individuals with mental illness typically wait at least four and a half years, with a maximum of 38 years, before seeking assistance from contemporary mental healthcare services (3). This delay in treatment may result in increased morbidity and mortality, as well as the emergence of several psychiatric and physical comorbidities and the use of life-threatening and life-altering self-treatments (such as licit and illicit substance misuse) (4).

Secondary prevention-oriented schizophrenia research has concentrated on specific early course durations, including the duration of untreated illness (DUI) and the duration of untreated psychosis (DUP). Early detection and early intervention are increasingly recognized as important factors for improving the course of schizophrenia (5, 6). The DUI is the time between the prodrome's development and the start of treatment. It emphasizes the crucial importance of early intervention in addressing any health condition, as delays in seeking or receiving treatment can lead to negative outcomes, whereas the DUP is most frequently thought of as the time between the onset of positive psychotic symptoms and the start of treatment (7).

The World Health Organization and the International Early Psychosis Association have emphasized that patients with first-episode psychosis should receive treatment within 3 months from the onset of symptoms to reduce the impact of the illness and increase the chance of recovery (8). However, the early symptoms of mental disorders are not always noticeable to lay people, and the seeking of treatment for psychosis is, therefore, sometimes delayed (9). Delayed treatment seeking is defined as the time interval between the onset of psychotic symptoms/syndrome and the commencement of adequate psychiatric treatment, even though the time interval qualifying this definition varies across studies (10). It highlights the potential barriers or obstacles that may result in delays in accessing or delivering appropriate care. Delays in the onset of treatment prolong the duration of untreated psychosis (DUP). A longer DUP is associated with poorer quality of life and treatment outcomes (11).

The prevalence of treatment-seeking delays for psychotic disorders was 49.7% with a treatment delay lasting more than 3 months (12) and 27.3% with a delay lasting more than 6 months (13). According to a study conducted in northern Ethiopia, 41.6% of patients have been delayed for more than 52 weeks ahead of psychiatric treatment (14).

The age ranged from 31 to 40 years, marital status, being single and divorced, unemployment, seeking psychiatric treatment after seeking religious treatment, perceiving mental illness as shameful, perceived stigma, societal attitudes, unawareness, and under-diagnosis are some of the modifiable and non-modifiable factors associated with delayed treatment seeking in some previous studies (14, 15).

Identifying the factors associated with delayed treatment-seeking will help predict and prevent longer durations of untreated psychosis in patient care. However, evidence of the magnitude and associated factors of delayed treatment is scarce in Ethiopia (3, 14). Therefore, the purpose of this study was to determine the magnitude of delayed treatment and its associated factors among people with schizophrenia spectrum disorders.

## Methods

### Study design and setting

A cross-sectional study was carried out at Dilla University Referral Hospital, southern Ethiopia, between June and September 2022. The hospital offers medical services to ~5 million people in the southern parts of Oromia, SNNPR, and Somalia and is situated 365 km south of the capital city, Addis Ababa. The psychiatry service was started in 1978 G.C., and ~530 patients attend mental health services monthly, with ~180 patients attending both inpatient and outpatient mental health services for schizophrenia and spectrum disorders, according to the hospital's HMIS report of mental health services.

### Populations

The source population consisted of all individuals with a DSM-5 diagnosis of schizophrenia spectrum disorders who received mental health care at Dilla University Referral Hospital in southern Ethiopia. The study population consisted of all adults (18 years and older) with schizophrenia spectrum disorders who received mental health treatment and were present at this facility during the data collection period. DSM-5 diagnoses of schizophrenia spectrum disorders in patients included in this study were confirmed by a senior mental professional specialist (Master of Science degree holder in Psychiatry) serving in Dilla University Referral Hospital.

### Sample size and sampling procedure

A sample size of 414 was calculated using a formula based on a 41.6% delayed treatment rate (14), a 95% confidence interval, a 5% margin of error, and a 10% non-response rate. A systematic random sampling method was used, with the sampling fraction (k) determined by dividing the total study subjects who received mental health treatment during the 5-month data collection period (850) by the required sample size (414). Participants were selected at every second interval until the final sample size was completed. The sampling frame was patient charts at both outpatient and inpatient departments.

### Study variables

The dependent variable was delayed treatment seeking, and the independent variables included various sociodemographic factors such as age, age at onset, sex, marital status, religion, educational status, economic status, place of residence, and distance to a healthy facility. Clinical and psychosocial factors such as social support, internalized stigma, self-esteem, resilience, family adaptability and cohesion, attitude toward psychiatric medication, perceived level of stress, and awareness about the availability of psychiatric treatment were also considered independent variables.

### Data collection and measurement tools

Face-to-face interviews using semi-structured questionnaires that included sociodemographic, clinical, and psychosocial factors were conducted to collect data. Data were collected by two psychiatry Bachelor of Science degree holders using Epicollect. Information was gathered from both the patient and their caregivers when the patient exhibited impaired insight. The data collection was supervised by a mental health Master of Science degree holder at the study site.

Delayed treatment seeking was determined based on the interview with the patient and their caregivers using a semi-structured questionnaire. Greater than the median of the total duration of untreated illness was considered delayed treatment seeking, and the same approach was used to determine the magnitude of delayed treatment seeking in a previous study conducted in Ethiopia (14).

Social support was measured using the Oslo Social Support Scale (OSSS-3) (16). The OSSS-3 total score ranges from 3–14. Scores from 3 to 8 are considered to indicate poor support; scores from 9 to 11 indicate intermediate support; and a score between 12 and 14 is considered to indicate strong social support. It has acceptable internal consistency (α = 0.640). This tool has also been used in Ethiopian settings (17–19).

FACES-III is a tool aimed at measuring three qualities of a family: family cohesion, family adaptability, and family type/functioning. To measure these qualities, Faces III utilizes ~20 questions: odd-numbered questions for cohesion and even-numbered questions for adaptability. Family type is determined by dividing the sum of the family cohesion and adaptability scores by two. Faces III boasts good internal consistency (*r* = 0.068) and high test-retest reliability (*r* = 0.080). However, in terms of validity, there is a low correlation between scales (*r* = 0.003). The scoring process for the tool is as follows: Family cohesion scores range from 10 to 50, with 10–34/1–2 indicating a disengaged family, 35–40/3–4 suggesting a separated family, 41–45/5–6 suggesting connectedness, and 46–50/7–8 indicating very connected families. Scores for family adaptability also range from 10 to 50, with 10–19/1–2 indicating rigidity, 20–24/3–4 indicating structured adaptability, 25–29/5–6 indicating flexibility, and 30–50/7–8 indicating very flexible families. Family type scores range from 1 to 8, with 1–2 indicating extreme family type, 3–4 indicating mid-range family type, 5–6 indicating moderately balanced families, and 7–8 indicating balanced families (20). Faces III has been used in Ethiopia (21, 22).

The 5-Question Stigma Indicator (5-QSI) is a tool consisting of five questions that was developed in 2017 in Uttar Pradesh, India. The aim of the tool is to assess and monitor neglected tropical disease (NTD)-related stigma based on the commonly used EMIC stigma scale. The 5-QSI-AP is used to evaluate and monitor feelings of stigma in affected people. In the 5-QSI, each item is scored based on the frequency of occurrence, with a score of 0 representing “never” or “I do not know,” a score of 1 representing “sometimes,” and a score of 2 representing “often/usually” (23). The total score ranges from 0 to 10, where a score over 5 is considered high internalized stigma, and a score under 5 is considered low internalized stigma.

Attitudes toward psychiatric medications were measured using a six-item questionnaire. The first four questions examined opinions about the benefits of these medications, such as aiding in the management of daily stress, improving relationships with loved ones, controlling symptoms, and promoting self-esteem. The remaining two questions focused on opinions about medication risks, including physical harm and interference with daily activities. Responses were rated on a 5-point Likert scale, ranging from “strongly agree” to “strongly disagree.” Scoring was conducted after reverse scoring the two risk questions, and the total score ranged from 6 to 30 (24). Scores of 18 or above were considered positive attitudes, while scores of 17 or lower indicated a negative attitude.

The Rosenberg Self-Esteem Scale (RSE) is a 10-item scale initially created to measure high school students' self-esteem. However, the scale has been utilized with various groups, including adults. The scale's coefficient of reproducibility is 0.92, signifying outstanding internal consistency. The test-retest reliability over 2 weeks shows correlations of 0.85 and 0.88, indicating excellent stability. The scale scores by adding up the individual 4-point items, with the negatively worded items being reverse-scored (25). The scale has been utilized in Ethiopia (26, 27).

Awareness about the availability of psychiatric treatment: To assess awareness of psychiatric treatment availability, a single item with possible answers of “yes” or “no” was used.

The perceived stress scale is a 10-item scale that measures perceived stress levels. The score ranges from 0 to 40, with scores of 0–13 considered low stress, 14–26 considered moderate stress, and 27–40 considered high stress (28). This scale has been utilized in Ethiopia (29, 30).

### Data quality control

The questionnaire was prepared in English and then translated into Amharic and Gedeuffa, which is the local language of the Gedeo Zone. To ensure consistency and understandability, the questionnaire was back-translated into English by two experts. Pre-testing was conducted using 5% of the sample size at Hawassa Comprehensive Specialized Hospital. Based on the feedback obtained from the pre-test, the final version of the questionnaire was developed. Data collectors and supervisors received training from the principal investigator on the questionnaire, data collection methods, quality control, and ethical considerations. The questionnaire was assessed for reliability and understanding. Supervision was conducted by site supervisors during data collection. After the data collection process, the completed questionnaires were checked for completeness and consistency.

### Data analyses

Data were collected using the Epicollect mobile application, which is a new platform for online and offline data collection. The collected data were exported to SPSS version 26 for analysis. Logistic regression analysis was used to assess the presence of an association between dependent and independent variables. Variables with a *p*-value < 0.25 in univariable logistic regression were entered into multivariable logistic regression. Statistical significance was considered at a *p*-value < 0.05, and the odds ratio with a 95% confidence interval estimated the strength of the association. Descriptive statistics, such as frequencies, percentages, and medians, were used to summarize the findings. Chi-square and odds ratios were calculated to determine the association between variables.

## Result

### Sociodemographic characteristics of participants

A total of 414 individuals with schizophrenia spectrum disorders were included in the study, resulting in a response rate of 100%. The participants had a median age of 33 years (interquartile range: 20–70 years). More than half were men (214; 51.7%), while most were married (188; 45.4%) and had not attended formal education (150; 36.3%). Regarding occupation, 156 (37.4%) of the participants were farmers. The majority hailed from urban areas (278; 67.1%), and most (228; 55.0%) had a daily income of more than 97 ETB (~US$1.9) (see Table 1).

**Table 1:** Description of socio-demographic characteristics of participants with schizophrenia spectrum disorders in Dilla University Referral Hospital, SNNPR, Ethiopia, 2022 (n = 414).

**Variables**	**Frequency**	**Percent**
**Gender**		
Male	214	51.7
Female	200	48.3
**Age**		
18–24	64	15.5
25–34	158	38.2
35–44	108	26.1
≥45	84	20.3
**Marital status**		
Single	120	29.0
Married	188	45.4
Divorced	62	15.0
Widowed	44	10.6
**Education status**		
Unable to read and write	150	36.3
Elementary	72	17.4
High school	74	17.8
Diploma and above	118	28.5
**Occupation status**		
Jobless	42	10.1
Farmer	156	37.7
Merchant	42	10.1
Government And Private Worker	71	17.1
Student	41	9.9
Retired	48	11.6
Other^*^	14	3.4
**Religious status**		
Protestant	175	42.3
Orthodox	109	26.3
Muslim	114	27.5
Other^**^	16	3.9
**Residency**		
Urban	278	67.1
Rural	136	32.9
**Distance to health service**		
≥5 km”	197	47.6
<5 km”	217	52.4
**Income status**		
≥97 ETB/day”	228	55.0
<97 ETB”	186	45.0

^*^Housewife, daily labor.

^**^Jehovah's Witness; ETB, Ethiopian birr.

### Clinical and psychosocial factors of participants

The study involved 414 participants, of whom 56.3% were diagnosed with schizophrenia disorder. The participants' median age at the onset of illness was 30, with an interquartile range of 16–55 years. Most had poor social support (48.3%) and a negative attitude toward psychiatric medication (66.6%). Over half had low levels of internalized stigma (57.2%) and low resilience levels (71.0%). Nearly all had a moderate perceived level of stress (94.2%). Most participants had no awareness of the availability of psychiatric treatment (51.7%) (see Table 2).

**Table 2:** Description of clinical and psychosocial factors of participants with schizophrenia spectrum disorders in Dilla University Referral Hospital, SNNPR, Ethiopia, 2022 (n = 414).

**Variables**	**Frequency**	**Percent**
**Clinical diagnosis**		
Schizophrenia	233	56.3
Schizophreniform	83	20.0
Schizoaffective disorder	8	1.9
Delusional disorder	16	3.9
Brief psychotic disorder	58	14.0
Other specified psychotic disorder	13	3.1
Psychotic disorder NOS	3	0.7
**Age of onset**		
<30	222	53.6
≥30	192	46.4
**Social support**		
Strong social support	120	28.9
Moderate social support	94	22.8
Poor social support	200	48.3
**Attitude toward psychiatric medication**		
Negative	276	66.6
Positive	138	33.4
**Internalized stigma**		
Low	220	53.1
High	194	46.9
**Resilience**		
Low resilience	294	71.0
Normal resilience	116	28.0
High resilience	4	1.0
**Perceived stress**		
Low stress	15	3.6
Moderate stress	390	94.2
High stress	9	2.2
**Awareness about availability of psychiatric treatment**		
Yes	200	48.3
No	214	51.7
**Attitude toward psychiatric medication**		
Positive	278	67.1
Negative	136	32.9

### Magnitude of delayed treatment seeking among people with schizophrenia spectrum disorders

**Figure 1:**
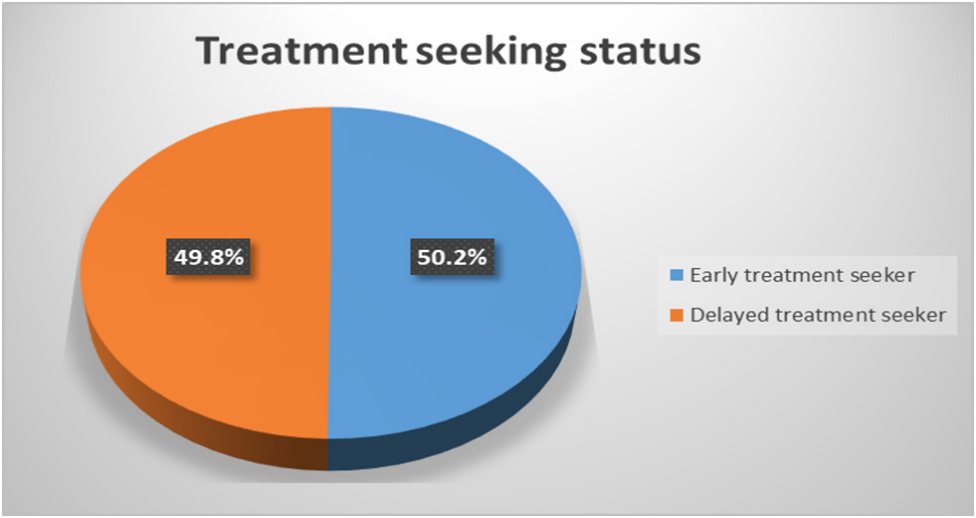
Rates of delayed treatment seeking among people with schizophrenia spectrum disorders in Dilla University Referral Hospital, SNNPR, Ethiopia, 2022.

In the current study, the median time taken by participants to seek modern treatment after the onset of schizophrenia spectrum disorders was 11 months, with an interquartile range of 1–207 months. This study found that 49.8% (95% CI = 44.9, 54.3) of people with schizophrenia spectrum disorders experienced delayed treatment seeking (see Figure 1).

### Factors associated with delayed treatment-seeking

This study found that several factors are associated with delayed treatment seeking, including distance from healthcare facilities, educational status, social support, family cohesion, and family adaptability. Participants who had a negative attitude toward psychiatric medication or lived more than 5 km from a health facility had 2.88 times [AOR = 2.88, 95%CI = (2.034, 4.469)] and 3.20 times [AOR = 3.20, 95%CI = (2.648, 5.251)] higher odds of delaying modern treatment, respectively, compared to their counterparts. In participants with higher education than just a diploma or high school, the chances of a delay in psychiatric treatment decreased by 96% [AOR = 0.040, 95%CI = (0.026, 0.077)] and 91% [AOR = 0.09, 95%CI = (0.071, 0.204)], respectively, when compared with those who did not attend formal education. Participants with poor social support, disengaged family cohesion, and rigid family adaptability were 3.97 times [AOR = 3.97, 95%CI = (2.999, 7.193)], 3.40 times [AOR = 3.40, 95%CI = (2.483, 6.273)], and 2.00 times [AOR = 2.00, 95%CI = (1.686, 4.044)] more likely to experience a delay in psychiatric treatment compared to their counterparts, respectively. Furthermore, those unaware of the availability of psychiatric treatment are 1.63 times [AOR = 1.63, 95%CI = (1.362, 2.626)] more likely to delay seeking it than those aware of its availability (see Table 3).

**Table 3:** Description of bivariable and multivariable binary logistic regression analysis showing an association between delayed treatment seeking and associated factors among participants with schizophrenia spectrum disorders in Dilla University Referral Hospital, SNNPR, Ethiopia, 2022.

**Variables**	**Category**	**Duration of untreated psychosis**		**Crude odds ratio (95%CI)**	**Adjusted odds ratio (95%CI)**	***P*-value**
		**Shorter**	**Longer**			
Age	18–24	28	36	1		
	25–34	78	70	0.69 (0.387, 1.259)	0.39 (0.425, 1. 425)	0.11
	35–44	60	48	0.62 (0.334, 1.160)	0.47 (0.369, 1.049)	0.06
	≥45	34	30	0.68 (0.342, 1.377)	0.37 (0.383, 1.231)	0.14
Gender	Female	140	60	1	1	
	Male	160	54	0.78 (0.511, 1.213)	0.61 (0.548, 1.132)	0.13
Distance from health facility	<5 km	137	80	1	1	
	≥5 km	62	135	3.48 (2.319, 5.216)	3.20 (2.648, 5.251)^*^	0.000
Attitude toward psychiatric medication	Positive	128	150	1		
	Negative	30	106	3.01 (1.886, 4.819)	2.88 (2.034, 4.469)^*^	0.000
Educational status	Unable to read and write	30	120	1	1	
	Elementary	52	20	0.096 (0.050, 0.185)	0.089 (0.056, 0.166)^*^	0.00
	High school	50	24	0.12 (0.064, 0.225)	0.09 (0.071, 0.204)^*^	0.00
	Diploma and above	100	18	0.045 (0.024, 0.085)	0.040 (0.026, 0.077)^*^	0.00
Social support	Strong social support	95	25	1	1	
	Moderate social support	76	18	0.90 (0.457, 1.771)	0.69 (0.510, 1.588)	0.39
	Poor social support	90	110	4.64 (2.758, 7.822)	3.97 (2.999, 7.193)^*^	0.00
Family cohesion	Very connected	80	50	1	1	
	Connected	60	45	1.20 (0.711, 2.026)	1.00 (0.773, 1.862)	0.25
	Separated	25	50	3.20 (1.763, 5.808)	2.60 (1.940, 5.277)^*^	0.0006
	Disengaged	30	74	3.95 (2.272, 6.855)	3.40 (2.483, 6.273)^*^	0.0001
Family adaptability	Very flexible	90	64	1	1	
	Flexible	50	30	0.84 (0.485, 1.469)	0.59 (0.530, 1.344)	0.27
	Structured	34	46	1.90 (1.101, 3.288)	1.50 (1.202, 3.011)^*^	0.019
	Rigid	25	75	2.61 (1.551, 4.398)	2.00 (1.686, 4.044)^*^	0.0001
Internalized stigma	Low	140	80	1		
	High	60	134	3.90 (2.594, 5.889)	3.24 (2.770, 5.514)^*^	0
Awareness about availability of psychiatric treatment	Yes	110	90	1		
	No	84	130	1.89 (1.279, 2.797)	1.63 (1.362, 2.626)^*^	0.000

Hosmer and Lemeshow test result was *P*-value = 0.069. ^*^indicate factors with significant association.

## Discussion

This study found that 49.8% of people with schizophrenia spectrum disorder experience delays in psychiatric treatments. Distance from health facilities, education, social support, family cohesion, and attitude affect treatment-seeking behavior. Lack of awareness about available psychiatric treatment also contributes to delays. Delayed treatment seeking was observed in the current study, which is consistent with findings from a previous study in Thailand (49.7%) (12). However, a study conducted in China showed a lower rate of 27.3% (13). This variation might be due to a difference in sample size. The result of this study aligns with a couple of studies in northern Ethiopia: one involving a total of 48% (31) participants from a multi-center study on relapse and associated factors among patients with schizophrenia spectrum disorder, and another reporting 41.6% (14) from a study conducted at Ayder Hospital in the Tigray region.

The current study found a lower prevalence of delayed psychiatric treatment compared to a previous study conducted to assess patterns of treatment-seeking behaviors for mental illnesses in southwest Ethiopia, which reported a delay of 65.1% (3). This could be attributed to increased awareness of mental illness and treatment options over the 11-year gap between studies.

Participants in this study who lived more than or equal to 5 km from a health facility were 3.2 times more likely to experience a delay in receiving psychiatric treatment. According to a systematic review, patients in low-income countries have to travel further to reach healthcare facilities due to a lack of providers and difficult terrain, making journeys even more challenging (32). Furthermore, a study in Rwanda found that a lack of geographical accessibility was a major barrier to mental health service utilization (33). These findings underscore the need for improved access to psychiatric treatments, especially in resource-constrained settings.

According to this study, participants with negative views about psychiatric medication are almost three times more likely to delay receiving psychiatric treatment. Similar findings have been observed in two additional studies conducted in Nigeria and southern Ethiopia (34, 35). Patients with psychotic disorders may also be suspicious about medication, thinking that it could cause harm or hearing voices telling them not to take it. Such negative attitudes can lead these patients to seek traditional or religious treatments, which can further delay psychiatric treatment for their condition (36).

The study found that participants with a higher educational status had a significantly lower chance of delaying psychiatric treatment than those with no formal education, with those with a diploma or higher experiencing a 96% reduction in delay odds. This finding is supported by a systematic review, which indicated that low education levels limit access to health care (32).

Poor social support was also linked to a higher likelihood of delaying treatment, with those lacking strong social support being almost four times as likely to delay seeking psychiatric treatment. Similar studies in Nigeria, Minnesota, and Tennessee reported a positive association between inadequate social support and poor utilization of mental health services (34) and delays in seeking medical care (37), respectively. These findings highlight the importance of education and social support for accessing timely psychiatric treatment.

Additionally, study participants with inflexible family adaptability and disengaged family cohesion had odds of experiencing delays in accessing modern treatment that were 3.40 times and 2.00 times higher, respectively, than those of their counterparts. This finding is consistent with a study conducted on the effects of patient personality traits and family cohesion on treatment delays among patients with first-episode schizophrenia spectrum disorder, which revealed that the delayed treatment group scored significantly lower on family cohesion and adaptability factors of the FACES-III (38). This implies that the family plays a crucial role in terms of patient access to mental health services when psychotic symptoms manifest.

Furthermore, as per this study, individuals participating in the research who exhibited elevated levels of internalized stigma had odds of encountering delays in psychiatric treatment that were 3.2 times higher compared with their counterparts. This finding is consistent with a study conducted in northern Ethiopia, which reported a positive association between stigma and delayed treatment seeking among mentally ill patients (14). It has been found that patients' preferences for sources of help are influenced by the stigma surrounding mental illness. According to a study from Nigeria, 20% of patients with mental illness were discouraged from accessing specialized care due to stigma and discrimination associated with their conditions (39). Intervention is crucial for this area since stigma can act as a major impediment to help-seeking behaviors and cause a delay in receiving psychiatric care.

Finally, in this study, the odds of delay in psychiatric treatment among study participants with no awareness of the availability of psychiatric treatment were 1.6 times higher when compared with their counterparts. This result was consistent with a study conducted in Rwanda, which mentioned a lack of awareness of the availability of mental health services as a factor contributing to delayed psychiatric treatment for patients with mental illness (33).

### Strengths and limitations of the study

This is a study on delayed treatment seeking and factors associated with it among patients with schizophrenia spectrum disorder. This study also assessed disengaged family cohesion, inflexible family adaptability, a lack of awareness of the availability of psychiatric treatment, and negative attitudes toward psychiatric treatment, among other factors that affect delayed psychiatric treatment. However, the study did not assess current substance use that might have an impact on delayed treatment seeking. Recall bias on the first onset of illness and/or the duration of illness could be a major limitation, as the majority might know the exact time and use only approximations. Using interviewer-administered questionnaires might have contributed to social desirability bias and the selection of participants from a single facility, which were other limitations of this study.

## Conclusion

The study's results showed a significant delay in seeking modern psychiatric treatment. Disengaged family cohesion, inflexible family adaptability, a lack of awareness of the availability of psychiatric treatment, high internalized stigma, and those with a negative attitude toward psychiatric treatment were significantly associated with delayed psychiatric treatment. Therefore, it is vital to provide interventions for factors that play a role in delayed psychiatric treatment. Several interventions can be implemented to address the factors contributing to delayed psychiatric treatment. Some of these are as follows:

1. Conduct awareness campaigns, workshops, or community outreach initiatives to educate people and families about mental health and the advantages of timely treatment. This can help dispel myths and reduce stigma.
2. Provide family-focused interventions aimed at improving family cohesion and flexibility through family counseling and support. Encourage open communication, understanding, and support systems to facilitate improved mental health care-seeking behavior.
3. Organize an anti-stigma campaign to dispel false beliefs and negative attitudes about receiving psychiatric treatment. Encourage individuals to share their recovery stories and promote positive messages about seeking help.
4. Integrate mental health services into primary healthcare facilities to increase the accessibility and availability of psychiatric services. This serves to overcome obstacles such as cost, distance, and time constraints.
5. Establish support groups or peer-led initiatives where people who have experienced mental health issues may share their stories, offer support, and act as role models for those seeking help.

By implementing these interventions, the goal is to mitigate the effects of disengaged family cohesion, inflexible family adaptability, low awareness, internalized stigma, and negative attitudes toward psychiatric treatment, ultimately leading to improved mental health outcomes for individuals.

## Data availability statement

The original contributions presented in the study are included in the article/supplementary material, further inquiries can be directed to the corresponding author.

## Ethics statement

Ethical approval was obtained from the Institutional Review Board (IRB) of Dilla University College of Medicine and Health Science before conducting the study. Participants, including patients and their families (in cases where patients did not have insight), were given informed consent after explaining the study's purpose. Personal identification was kept confidential throughout the study. Participants were also assured that they could withdraw from the study at any point if they wished.

## Author contributions

MN and BT prepared the manuscript for publication and participated in writing, revising, or critically evaluating the article, gave final approval for the version to be published, agreed to the journal's submission, and accepted responsibility for all aspects of the work. All authors significantly contributed to the published article, including the generation of ideas, study design, execution, data collection, analysis interpretation, contributed to the article, and approved the submitted version.
